# Chemical Composition and Anti-Inflammatory Effect of Ethanolic Extract of Brazilian Green Propolis on Activated J774A.1 Macrophages

**DOI:** 10.1155/2013/976415

**Published:** 2013-06-06

**Authors:** Ewelina Szliszka, Alicja Z. Kucharska, Anna Sokół-Łętowska, Anna Mertas, Zenon P. Czuba, Wojciech Król

**Affiliations:** ^1^Department of Microbiology and Immunology, Medical University of Silesia in Katowice, Jordana 19, 41 808 Zabrze, Poland; ^2^Department of Fruit and Vegetables and Cereals Technology, Wrocław University of Environmental and Life Sciences, Chełmońskiego 37/41, 51 630 Wrocław, Poland

## Abstract

The aim of this study was to investigate the chemical composition and anti-inflammatory effect of ethanolic extract of Brazilian green propolis (EEP-B) on LPS + IFN-**γ** or PMA stimulated J774A.1 macrophages. The identification and quantification of phenolic compounds in green propolis extract were performed using HPLC-DAD and UPLC-Q-TOF-MS methods. The cell viability was evaluated by MTT and LDH assays. The radical scavenging ability was determined using DPPH^•^ and ABTS^•+^. ROS and RNS generation was analyzed by chemiluminescence. NO concentration was detected by the Griess reaction. The release of various cytokines by activated J774A.1 cells was measured in the culture supernatants using a multiplex bead array system based on xMAP technology. Artepillin C, kaempferide, and their derivatives were the main phenolics found in green propolis. At the tested concentrations, the EEP-B did not decrease the cell viability and did not cause the cytotoxicity. EEP-B exerted strong antioxidant activity and significantly inhibited the production of ROS, RNS, NO, cytokine IL-1**α**, IL-1**β**, IL-4, IL-6, IL-12p40, IL-13, TNF-**α**, G-CSF, GM-CSF, MCP-1, MIP-1**α**, MIP-1**β**, and RANTES in stimulated J774A.1 macrophages. Our findings provide new insights for understanding the anti-inflammatory mechanism of action of Brazilian green propolis extract and support its application in complementary and alternative medicine.

## 1. Introduction

Propolis is a resinous substance collected by honeybees from the leaf bud and bark of certain plants. *Baccharis dracunculifolia*, *Eucalyptus citriodora*, *Araucaria angustifolia*, and *Myrocarpus frondosus* are major botanical sources of propolis from southeast Brazil (States of Minas Gerais and Sao Paulo) called due to its colour green propolis [1, 2]. Extracts of Brazilian green propolis possess antioxidant, antimicrobial, anti-inflammatory, chemopreventive, and anticancer properties [3–9]. Therefore, propolis has been extensively used in food, beverages, and dietary supplements to improve health and prevent diabetes, cancer, inflammatory, and heart diseases [10–13]. The medical application of propolis has led to increased interest in its chemical composition depending on the geographical origin and specific flora of the region, seasonality or methods of harvesting the raw material [1–4].

Our study was designed to investigate the chemical composition and anti-inflammatory effect of ethanolic extract of Brazilian green propolis (EEP-B) on LPS (lipopolysaccharide) + IFN-*γ* (interferon *γ*) or PMA (phorbol 12-myristate 13-acetate) stimulated J774A.1 macrophages. 

Macrophages are the first effector cells of the immune system that protect against microbial infection and tissues injury. Macrophages have three important functions: antigen presentation, phagocytosis, and synthesis of various inflammatory mediators [14, 15]. Propolis affects the nonspecific immunity *via* modulation of macrophages activity [3, 13]. During inflammation, upon stimulation by LPS from Gram-negative bacteria and IFN-*γ* from host immune cells, macrophages release large amounts of reactive oxygen (ROS) and nitrogen species (RNS), nitric oxide (NO), and numerous cytokines [16]. Overexpression of these mediators causes pathological, acute, or chronic inflammatory responses [14].

This is the first comprehensive report explaining an anti-inflammatory potential of Brazilian green propolis extract on macrophage *in vitro* model. We determined the effect of EEP-B on production of NO, ROS, RNS, and cytokines: interleukin (IL)-IL-1*α*, IL-1*β*, IL-3, IL-4, IL-5, IL-6, IL-9, IL-10, IL-12p40, IL-13, IL-17, TNF-*α* (tumor necrosis factor *α*), IFN-*γ* (interferon *γ*), G-CSF (granulocyte colony-stimulating factor), GM-CSF (granulocyte-macrophage colony-stimulating factor), MCP-1 (monocyte chemotactic protein 1), MIP-1*α* (macrophage inflammatory protein 1*α*), MIP-1*β* (macrophage inflammatory protein 1*β*), and RANTES (regulated upon activation normal T cell expressed and secreted) in activated J774A.1 cells.

It has been suggested that the immunomodulatory properties of propolis is contributed by its flavonoids and phenolic acids [3, 5, 9, 17]. The typical ingredients of Brazilian green propolis are kaempferide (3,5,7-trihydroxy-4′-methoxyflavone) and cinnamic acid derivatives: *p*-coumaric acid (4-hydroxycinnamic acid), artepillin C (3,5-diprenyl-4-hydroxycinnamic acid), baccharin (3-prenyl-4-(dihydrocinnamoyloxy)-cinnamic acid) and drupanin (3-prenyl-4-hydroxycinnamic acid) [1, 4, 18]. We performed the detailed identification and quantification of phenolic compounds in propolis extract. 

The present study analyzed chemical composition of green propolis and confirmed its significant role in suppressing chronic inflammation and reducing risk of related human health problems. Our findings provide new insights for understanding the anti-inflammatory mechanism of action of Brazilian green propolis extract and support its application in complementary and alternative therapies.

## 2. Materials and Methods

### 2.1. General

The Brazilian green propolis sample was kindly supplied by Nihon Natural Foods Co., Ltd. (Tokyo, Japan). LPS (LPS *E. coli* O111:B4) was obtained from Fluka Chemie GmbH (Buchs, Switzerland) and recombinant mouse IFN-*γ* was purchased from R&D Systems (Minneapolis, MN, USA). PMA, DMSO (dimethyl sulphoxide), acetonitrile, and formic acid were obtained from Sigma-Aldrich (Steinheim, Germany). Acetonitrile for LC-MS was purchased from POCh (Gliwice, Poland). The following compounds were used as standards: sakuranetin, hesperitin, caffeic acid (Roth, Karlsruhe, Germany), kaempferol, pinocembrin, naringenin, *p*-coumaric acid (Sigma-Aldrich, Steinheim, Germany), kaempferide, ferulic acid (Serva, Heidelberg, Germany), isosakuranetin (TransMIT GmbH, Giessen, Germany), and artepillin C (Wako Pure Chemicals, Osaka, Japan).

### 2.2. Preparation of Brazilian Green Propolis Extract

The Brazilian green propolis sample was collected manually from beehive located in southeast Brazil (the state of Minas Gerais) and was kept desiccated prior to processing. The sample was extracted in 95% v/v ethyl alcohol, in a hermetically sealed glass vessel for 4 days at 37°C, under occasional shaking. The ethanolic extract of Brazilian green propolis (EEP-B) was then filtered through Whatman filter paper no. 4 and evaporated in a rotary evaporator, under reduced pressure at 60°C. The same collection and extraction procedures were used throughout all our laboratory studies [6, 12]. EEP-B was dissolved in DMSO (50 mg/mL), and the final concentration of DMSO in the culture medium was controlled at 0.1% (v/v). 

### 2.3. Identification and Quantification of Phenolic Compounds by HPLC-DAD Method

Phenolic compounds were determined using Dionex (USA) HPLC system equipped with diode array detector model Ultimate 3000, a quaternary pump LPG-3400A, autosampler EWPS-3000SI, and thermostated column compartment TCC-3000SD and controlled by Chromeleon v.6.8 software. The reversed phase Cadenza 5CD-C18 (75 mm × 4.6 i.d.) column (Imtakt, Kyoto, Japan) with guard column Cadenza (5 × 4.6 i.d.) guard column (Imtakt, Kyoto, Japan) was used. The mobile phase was composed of (A) 0.1% (v/v) formic acid in water and (B) acetonitrile. The applied elution conditions were 0 min 20% B; 0–10 min linear gradient from 20% to 30% B; 10–40 min linear gradient from 30% to 40% B; 40–60 min, linear gradient from 40% to 60% B; 60–80 min, linear gradient from 60% to 80% B; and then again the initial conditions [19–23]. The flow rate was 1 mL/min, and the injection volume was 20 *μ*L. The column was operated at 30°C. The compounds were monitored at 290 nm, 325 nm, and 370 nm.

### 2.4. Identification and Quantification of Phenolic Compounds by LC-MS Method

Compounds identification was performed on an Acquity ultra-performance liquid chromatography (UPLC) system coupled with a quadruple time of flight (Q-TOF) MS instrument (UPLC/Synapt Q-TOF MS, Waters Corp., Milford, MA, USA) with an electrospray ionization (ESI) source. Separation was achieved on the Acquity BEH C18 column (100 mm × 2.1 mm i.d., 1.7 *μ*m; Waters). Detection wavelengths were set at 290, 325, and 370 nm. A mobile phase was a mixture of 1.5% formic acid (A) and acetonitrile (B). The gradient program was as follows: initial conditions-95% (A), 12 min-5% (A), 13 min-5% (A), 14.5 min-95% (A), and 16 min-95% (A). The flow rate was 0.45 mL/min and the injection volume was 5 *μ*L. The column was operated at 30°C. The major operating parameters for the Q-TOF MS were set as follows: capillary voltage 2.0 kV, cone voltage 45 V, cone gas flow of 11 L/h, collision energy 50 eV, source temperature 100°C, desolvation temperature 250°C, collision gas, argon; desolvation gas (nitrogen) flow rate, 600 L/h; data acquisition range, *m*/*z* 100–1.000 Da; ionization mode, negative [24–26]. The data were collected by Mass-Lynx V 4.1 software.

### 2.5. Cell Culture

Murine macrophage J774A.1 cell line was obtained from ATCC (American Type Culture Collection, Manassas, VA, USA). Cells were cultured in Dulbecco's modified Eagle's medium supplemented with 10% heat-inactivated fetal bovine serum, 100 U/mL penicillin, and 100 *μ*g/mL streptomycin at 37°C and 10% CO_2_ in a humidified incubator [27]. Reagents for cell culture were purchased from ATCC. J774A.1 cells were seeded at a density of 1 × 10^6^/mL cells (2 × 10^5^/well) in 96-well plates at the presence of LPS (200 ng/mL) and IFN-*γ* (25 U/mL) with or without EEP-B for 24 h.

### 2.6. Cell Viability Assay

The cell viability was determined by the 3-(4,5-dimethyl-2-thiazyl)-2,5-diphenyl-2*H*-tetrazolium bromide (MTT) reduction assay as described [28, 29]. This test is based on the cleavage of the tetrazolium salt MTT to a blue formazan dye by viable cells. The J774A.1 cells (1 × 10^6^/mL) were seeded 4 h before the experiments in a 96-well plate. EEP-B at the concentrations of 5–50 *μ*g/mL with or without LPS + IFN-*γ* was added to the cells. The final volume was 200 *μ*L. After 24 h the medium was removed and 20 *μ*L MTT solutions (5 mg/mL) (Sigma Chemical Company, St. Louis, MO, USA) were added to each well for 4 h. The resulting formazan crystals were dissolved in DMSO. The controls included native cells and medium alone. The spectrophotometric absorbance was measured at 550 nm wavelength using a microplate reader (ELx 800, Bio-Tek Instruments Inc., Winooski, VT, USA). The cytotoxicity as percentage of cell death was calculated by the formula: (1 − [absorbance of experimental wells/absorbance of control wells]) × 100%.

### 2.7. Cytotoxicity Assay

The cytotoxicity of EEP-B was determined by using LDH activity assay kit (Roche Diagnostics GmbH, Mannheim, Germany) [30, 31]. Lactate dehydrogenase (LDH) is a stable cytosolic enzyme released upon membrane damage in necrotic cells. The J774A.1 (1 × 10^6^/mL) cells were treated with 5–50 *μ*g/mL EEP-B with or without LPS + IFN-*γ* for the indicated period of time. LDH released in culture supernatants is detected with coupled enzymatic assay, resulting in the conversion of a tetrazolium salt into a red formazan product. The maximal release of LDH was obtained after treating control cells with 1% Triton X-100 (Sigma Chemical Company, St. Louis, MO) for 10 min at room temperature. The spectrophotometric absorbance was measured at 490 nm wavelength using a microplate reader (ELx 800, Bio-Tek Instruments Inc., Winooski, VT, USA). The percentage of necrotic cells was expressed using the following formula: (sample value/maximal release) × 100%.

### 2.8. DPPH Radical Scavenging Activity

Hydrogen-donating activity was measured using 1,1-diphenyl-2-picrylhydrazyl radical (DPPH^•^) (Sigma Chemical Company, St. Louis, MO) following a previously reported protocol [32]. EEP-B (0.1 mL) was mixed with 0.9 mL of 0.041 mM DPPH^•^ in ethanol and stored at room temperature in the dark for 30 min. The absorbance of the resulting solutions was measured at 517 nm wavelength using V-630 Spectrophotometer (Jasko International Co., Tokyo, Japan). The percentage of scavenging activity was calculated by the formula: DPPH^•^ scavenging activity = 1 − (absorbance of experimental wells/absorbance of control wells) × 100%. The scavenging activity of the sample was expressed as the ED_50_ value, the concentration required to scavenge 50% of DPPH^•^. Ascorbic acid was used as a standard.

### 2.9. ABTS Cation Radical Scavenging Activity

2,2′-Azinobis(3-ethylbenzothiazoline-6-sulfonic acid) radical cation (ABTS^•+^) (Sigma Chemical Company, St. Louis, MO) scavenging activity was determined according to the previously described procedure [32]. EEP-B (0.1 mL) was mixed with potassium phosphate buffer (0.1 mL of 0.1 M) and hydrogen peroxide (10 *μ*L of 10 mM) and preincubated at 37°C in the dark for 5 min. Next, ABTS (30 *μ*L of 1.25 mM in 0.05 M phosphate-citrate buffer) and peroxidase (30 *μ*L of 1 unit/mL) were added to the mixture and then incubated at 37°C in the dark for 10 min. The absorbance of the resulting solutions was measured at 417 nm wavelength using V-630 Spectrophotometer (Jasko International Co., Tokyo, Japan). The percentage of scavenging activity was calculated by the formula: ABTS^•+^ scavenging activity = 1 − (absorbance of experimental well/absorbance of control wells) × 100%. The scavenging activity of the sample was expressed as the ED_50_ value, the concentration required to scavenge 50% of ABTS^•+^. Ascorbic acid was used as a standard.

### 2.10. Detection of ROS and RNS Production by Chemiluminescence

The chemiluminescence of J774A.1 macrophages was evaluated by microplate method in Hank's balanced salt solution, pH 7.4, at room temperature. The cells were incubated with 0.01–10 *μ*g/mL EEP-B for 30 min. Next, luminol (Sigma Chemical Company, St. Louis, MO, USA) solution was added to wells containing 2 × 10^5^ cells, giving a final concentration of 110 *μ*M. After 5 min, for macrophages stimulation PMA solution was injected to obtain the concentration of 0.8 *μ*M. The final volume of each sample was 200 *μ*L. The chemiluminescence was determined for 5 min with luminol alone and after stimulation with PMA for 30 min. The measuring system was equipped with LB 960 CentroXS^3^ microplate luminometer (Berthold Technologies GmbH, Wildbad, Germany) [33].

### 2.11. Quantification of NO Production

J774A.1 macrophages (1 × 10^6^/mL) stimulated with LPS + IFN-*γ* were incubated with 5–50 *μ*g/mL EEP-B for 24 h. After this time NO production was determined by measuring the accumulation of nitrite, a stable end product, in the culture supernatant according to the Griess reaction [27, 34]. Equal volumes of culture supernatant from each well or medium (100 *μ*L) were mixed with 100 *μ*L of Griess reagent in a 96-well plate and incubated for 15 min at room temperature. The spectrophotometric absorbance was read at 550 nm wavelength in Eon Microplate Spectrophotometer (BioTek, Winooski, VT, USA) and the nitrite concentration in the medium was calculated using sodium nitrite as a standard. Nitrite was not detectable in cell-free medium.

### 2.12. Multiplex Bead-Based Cytokine Assay

Cytokines released from J774A.1 macrophages treated with EEP-B were determined in the cell culture supernatants with a Pro Mouse Cytokines 19-plex assay kit for IL-1*α*, IL-1*β*, IL-3, IL-4, IL-5, IL-6, IL-9, IL-10, IL-12p40, IL-13, IL-17, TNF-*α*, IFN-*γ*, G-CSF, GM-CSF, MCP-1, MIP-1*α*, MIP-1*β*, and RANTES (Bio-Rad Laboratories Inc., Hercules, CA, USA). This test was performed using Bio-Plex 200 System based on xMAP suspension array technology (Bio-Rad Laboratories Inc., Hercules, CA, USA). The LPS + IFN-*γ* stimulated and native J774A.1 cells (1 × 10^6^/mL) were incubated with or without 25–50 *μ*g/mL EEP-B for 24 h. Standard curves for each cytokine were generated using kit-supplied reference cytokine sample. The assay is designed for the multiplexed quantitative measurement of multiple cytokines in a single well using 50 *μ*L of sample. Briefly, the following procedure was performed: after prewetting the 96-well filter plate with washing buffer, the solution in each well was aspirated using a vacuum manifold. Next, the cell culture supernatants were incubated with antibody-conjugated beads for 30 min. Following the incubational period, detection antibodies and streptavidin-PE were added to each well for 30 min. Then, after washing with buffer to remove the unbound streptavidin-PE, the beads bound to each cytokine were analyzed in the Bio-plex Array Reader (Bio-Plex 200 System). The fluorescence intensity was evaluated using Bio-Plex Manager software (Bio-Rad) [33, 35].

### 2.13. The Statistical Analysis

The values represent mean ± SD of two, three, or four independent experiments performed in duplicate or quadruplicate. Significant differences were analyzed using Student's *t*-test and *P*-values <0.05 were considered significant. The concentration-response curves were analyzed using Pharma/PCS version 4 (Pharmacological Calculations System) software.

## 3. Results

### 3.1. The Content and Characterization of Phenolic Compounds Identified in Brazilian Green Propolis Extract

**Figure 1:**
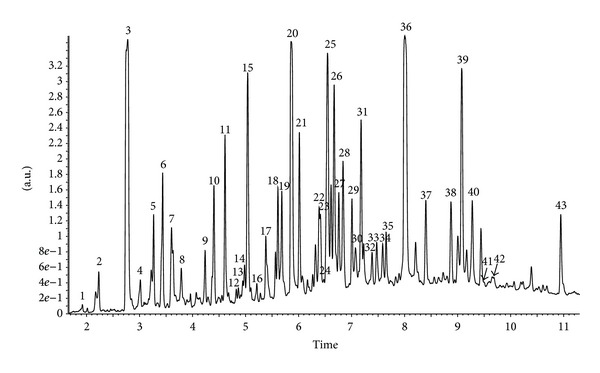
UPLC-DAD chromatogram (290 nm) of compounds of ethanol extract from Brazilian green propolis.

**Figure 2:**
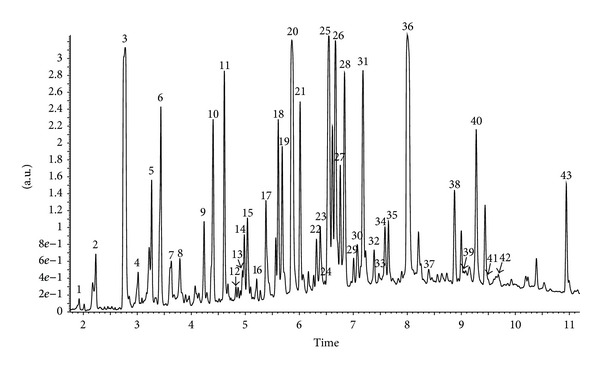
UPLC-DAD chromatogram (325 nm) of compounds of ethanol extract from Brazilian green propolis.

**Figure 3:**
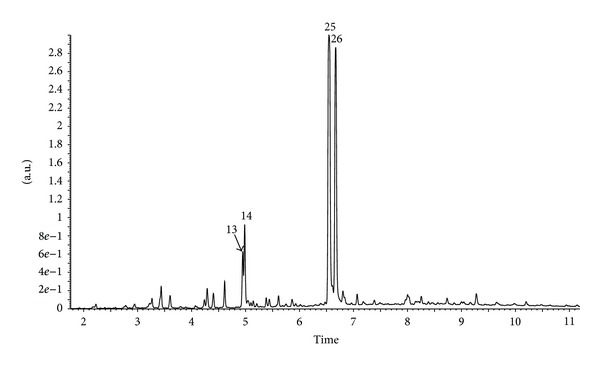
UPLC-DAD chromatogram (370 nm) of compounds of ethanol extract from Brazilian green propolis.

**Figure 4:**
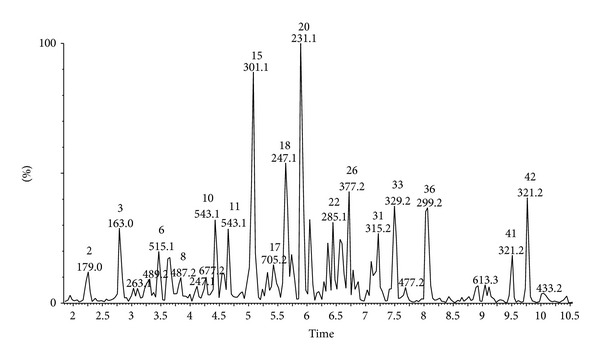
UPLC-ESI-MS (negative ion) chromatogram of main compounds of ethanol extract from Brazilian green propolis.

The identification and quantification of phenolic compounds in Brazilian green propolis extract were performed using HPLC-DAD and UPLC-Q-TOF-MS methods. Qualitative analysis results obtained by LC-ESI/MS methods and quantitative analysis data obtained by HPLC (quantified using DAD detection) are presented in Figures 1, 2, 3, and 4 and Table 1. A total of forty-three phenolic ingredients were found in tested propolis sample. Thirty-four compounds were identified by comparison of their UV and MS/MS spectra to standards and/or to the literature data, whereas another nine compounds remained unknown. Kaempferide, with its derivatives, and hesperitin, which were characterized by MS from their molecular ions at *m*/*z* 299.0572 and 301.0709, respectively, are the major flavonoids identified in Brazilian green propolis. Among the phenolic acids, prevailed *p*-coumaric acid (*m*/*z* 163.0406 and fragment at *m*/*z* 119 resulting from the loss of a COO group) and prenylated cinnamic acid derivatives: artepillin C (*m*/*z* 299.1634), baccharin (*m*/*z* 363.1619), and drupanin (*m*/*z* 231.1025) (Table 1).

### 3.2. Effect of Brazilian Green Propolis Extract on Viability of J774A.1 Macrophages

**Figure 5:**
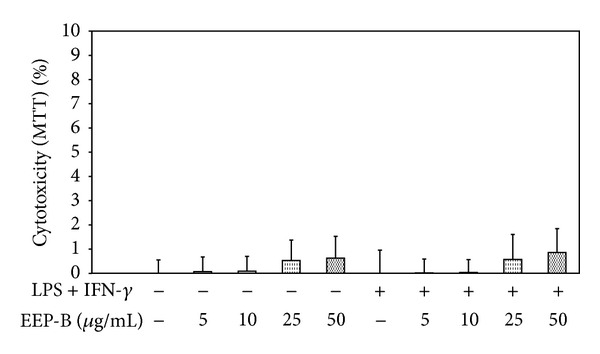
Effect of EEP-B on viability of J774A.1 macrophages. The cytotoxicity was evaluated by MTT assay after 24 h incubation of J774A.1 cells with 5–50 *μ*g/mL EEP-B and/or LPS + IFN-*γ*. The values represent mean ± SD of three independent experiments (*n* = 12).

The cell viability in the presence of 5–50 *μ*g/mL EEP-B and/or LPS + IFN-*γ* for 24 h was measured by MTT test (Figure 5). The cytotoxicity of the propolis extract at the same concentrations and incubation time was evaluated by LDH assay. EEP-B at the concentrations of ≤50 *μ*g/mL did not influence the cell viability and did not exert cytotoxic effect. Therefore for further studies of anti-inflammatory properties EEP-B was used at the concentrations of 5–50 *μ*g/mL.

### 3.3. Antioxidant Activity of Brazilian Green Propolis Extract

Antioxidant activity of EEP-B was investigated by using two different methods for stable DPPH^•^ and ABTS^•+^. The propolis extract exhibited strong scavenging potential against DPPH^•^ (ED_50_ of 24.1 *μ*g/mL) and ABTS^•+^ (ED_50_ of 40.6 *μ*g/mL) compared with ascorbic acid (ED_50_ of 15.8 *μ*g/mL and 10.1 *μ*g/mL, resp.).

### 3.4. Effect of Brazilian Green Propolis Extract on ROS and RNS Production in PMA Stimulated J774A.1 Cells

**Figure 6:**
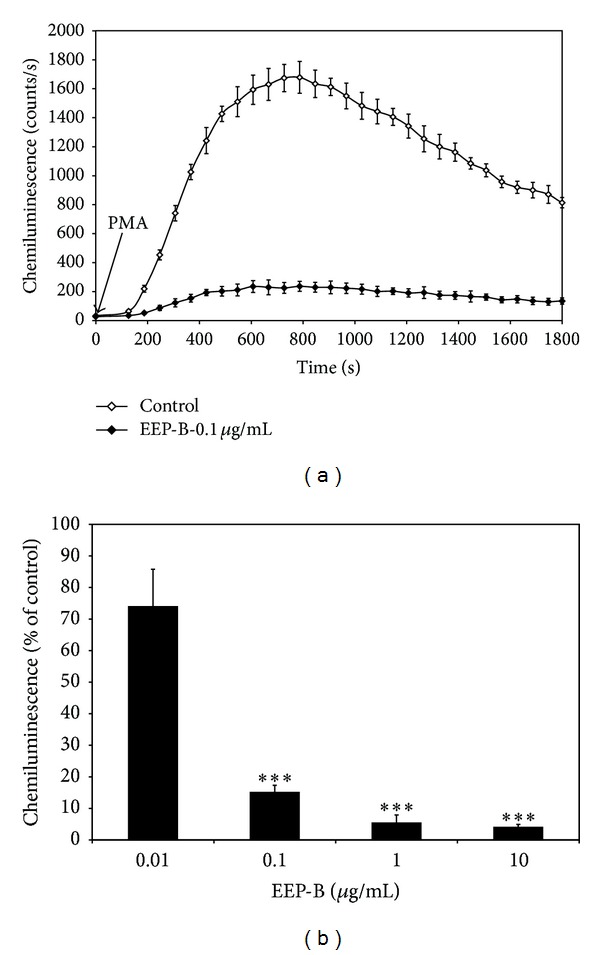
Effect of EEP-B on chemiluminescence of PMA activated J774A.1 macrophages: (a) time course of chemiluminescence; (b) chemiluminescence of PMA activated J774A.1 macrophages treated with 0.01–10 *μ*g/mL EEP-B for 30 min. Chemiluminescence was determined using microplate luminometer and expressed as a percentage of the PMA-stimulated cells. The values represent mean ± SD of four independent experiments (*n* = 8) *** = *P* < 0.001 compared to PMA-stimulated cells.

Changes in production of ROS and RNS in macrophages were determined by chemiluminescence assay. EEP-B at the concentrations of 0.01–10 *μ*g/mL suppressed the chemiluminescence in PMA stimulated J774A.1 cells in dose-dependent manner with an ED_50_ of 0.02 *μ*g/mL (Figure 6).

### 3.5. Effect of Brazilian Green Propolis Extract on NO Production in LPS + IFN-*γ* Stimulated J774A.1 Cells

**Figure 7:**
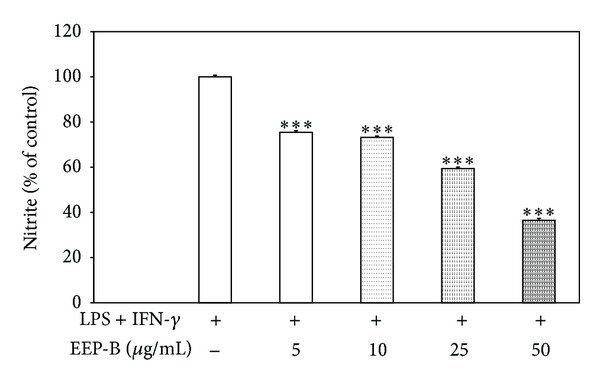
Effect of EEP-B on nitrite (NO) production in LPS + IFN-*γ* stimulated J774A.1 macrophages. J774A.1 cells were incubated with 5–50 *μ*g/mL EEP-B and/or LPS + IFN-*γ* for 24 h. NO production was measured by the Griess reaction assay and expressed as a percentage of LPS + IFN-*γ*-stimulated cells. The values represent mean ± SD of three independent experiments (*n* = 12) *** = *P* < 0.001 compared to LPS + IFN-*γ*-stimulated cells.

NO production was determined by measuring the accumulation of nitrite in the culture supernatants using Griess reagent. After 24 h LPS + IFN-*γ* stimulation nitrite concentration markedly increased, but LPS + IFN-*γ*-induced NO synthesis in J774A.1 cells was significantly decreased by EEP-B in dose-dependent manner (ED_50_ = 30.1 *μ*g/mL). The inhibitory effect of 5–50 *μ*g/mL EEP-B on NO production in LPS + IFN-*γ* stimulated macrophages is presented in Figure 7. The propolis extract did not interfere with the viability of J774A.1 cells, as shown in MTT and LDH test. The ED_50_ value of the EEP-B within the nontoxic concentration range suggests that the inhibition of nitrite accumulation was specific to responses by macrophages (due to inhibitory activity on NO production and not cytotoxic property of EEP-B).

### 3.6. Effect of Brazilian Green Propolis Extract on Cytokine Production in LPS + IFN-*γ* Stimulated J774A.1 Cells

**Figure 8:**
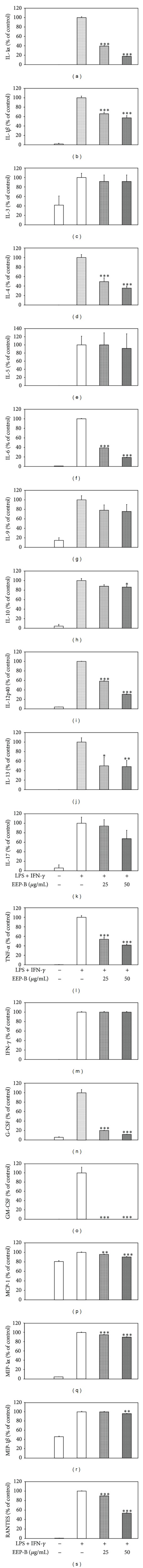
Effect of EEP-B on cytokines production in LPS + IFN-*γ* stimulated J774A.1 macrophages: (a) IL-1*α*, (b) IL-1*β*, (c) IL-3, (d) IL-4, (e) IL-5, (f) IL-6, (g) IL-9, (h) IL-10, (i) IL-12p40, (j) IL-13, (k) IL-17, (l) TNF-*α*, (m) IFN-*γ*, (n) G-CSF, (o) GM-CSF, (p) MCP-1, (q) MIP-1*α*, (r) MIP-1*β*, and (s) RANTES. J774A.1 cells were incubated with 25–50 *μ*g/mL EEP-B and/or LPS + IFN-*γ* for 24 h. Cytokine concentrations in the culture medium were determined by Multiplex (19-plex) bead-based cytokine assay. The values represent mean ± SD of two independent experiments (*n* = 8) * = *P* < 0.05, ** = *P* < 0.01, *** = *P* < 0.001 compared to LPS + IFN-*γ*-stimulated cells.

The effect of EEP-B on production of cytokine IL-1*α*, IL-1*β*, IL-3, IL-4, IL-5, IL-6, IL-9, IL-10, IL-12p40, IL-13, IL-17, TNF-*α*, IFN-*γ*, G-CSF, GM-CSF, MCP-1, MIP-1*α*, MIP-1*β*, and RANTES in LPS + IFN-*γ* stimulated J774A.1 cells is shown in Figure 8. Specifically, native and activated J774A.1 macrophages were treated with 25–50 *μ*g/mL EEP-B for 24 h. The cytokines released in culture supernatants were analyzed simultaneously by Bio-plex Suspension Array System. This assay is designed for the multiplexed quantitative measurement of cytokines (19-plex) in a single well using 50 *μ*L of sample. EEP-B significantly decreased synthesis of IL-1*α*, IL-1*β*, IL-4, IL-6, IL-12p40, IL-13, TNF-*α*, G-CSF, GM-CSF, MCP-1, MIP-1*α*, MIP-1*β*, and RANTES in LPS + IFN-*γ* stimulated J774A.1 cells in dose-dependent manner. The EEP-B did no influence the concentrations of IL-3, IL-5, IL-9, IL-17, and IFN-*γ* and only slightly downregulates IL-10 in culture supernatants derived from activated macrophages.

## 4. Discussion

In Brazil, twelve distinct groups of propolis have been classified according to their botanical origin and biological properties. Green propolis collected in southern region of Brazil (States of Minas Gerais and Sao Paulo) belongs to Group 12 (propolis G12) [1]. Chemical evidence suggested that *Baccharis dracunculifolia* is the main plant source for green propolis [36]. Park et al. demonstrated similar profiles of phenolic components identified in green propolis extract and *Baccharis dracunculifolia* resins [37]. The tested sample of green propolis was rich in hesperitin, kaempferide and its derivatives, and cinnamic acid derivatives: *p*-coumaric acid, artepillin C, baccharin, and drupanin. Our studies confirmed the results obtained by Park et al. [1, 37] and Banskota et al. [38].

Macrophages are the main cells responsible for innate (nonspecific) immunity. The J774A.1 cells, a murine macrophage cell line, are widely used to establish inflammatory model *in vitro* [27]. Numerous findings showed antioxidant and anti-inflammatory effects of propolis extract [3, 4, 13, 18, 21, 27, 39, 40]. The biologically active molecules in green propolis are phenolic acids and flavonoids, which act as scavengers of free radicals and inhibitors of nitric oxide and inflammatory cytokines production by macrophages and/or neutrophils [4, 13, 16–18, 27, 34, 39, 40]. Kaempferide, artepillin C, and their derivatives are the major constituents identified in our tested sample of Brazilian green propolis extract. 

Brazilian green propolis exhibits significant antioxidant properties by scavenging ROS and inhibiting chemiluminescence reactions [4, 18, 40]. The topical or oral treatment of animals with Brazilian propolis extracts demonstrated potential effect against oxidative stress [41]. DPPH^•^ and ABTS^•+^ tests have been widely used for evaluating antioxidant properties of natural phenolic compounds. Antioxidants intercept the free radical chain oxidation by donating hydrogen from the phenolic hydroxyl groups, thereby forming stable end products, which do not initiate or propagate further oxidation. EEP-B exerted strong free radical scavenging activity based on reduction of DPPH^•^ and ABTS^•+^ [4, 17]. Izuta et al. showed the ability of scavenging DPPH^•^ by green propolis and artepillin C, in contrast to other prenylated derivatives of cinnamic acid, drupanin and baccharin, which did not cause similar effect. The findings indicate that 3-prenyl chain of cinnamic acid is important for antioxidant activity of artepillin C [42]. Hayashi et al. noticed strong antioxidant effect of two compounds isolated from Brazilian propolis, kaempferide, and artepillin C [43]. In the present study we verified potent scavenging capability of EEP-B against DPPH^•^ and ABTS^•+^.

Regarding the anti-inflammatory property, the effect of EEP-B in ROS and RNS scavenging was also determined by chemiluminescence assay. We evaluated for the first time the role of green propolis in the oxidative metabolism of PMA stimulated macrophages. The ROS and RNS release by activated J774A.1 cells was significantly inhibited by EEP-B in dose-dependent manner. Król et al. and Simões et al. proved that kaempferide and extracts of Polish and Brazilian green propolis decrease the chemiluminescence produced by stimulated neutrophils [18, 39, 40].

NO, a short half-life free radical, is an effector molecule in host defense against pathogens. NO production in macrophages is mediated by the inducible nitric oxide synthase (iNOS). However, excessive release of NO induced by LPS, IFN-*γ*, or TNF-*α* has detrimental effects on many organ systems of the body, leading to acute or chronic inflammatory diseases [44]. Song et al. reported that treatment of RAW264.7 cells with extract of Korean propolis markedly suppresses NO production and iNOS mRNA and protein expression induced by LPS + IFN-*γ* [45]. Blonska et al. observed the inhibition of NO synthesis and iNOS mRNA expression in LPS stimulated J774A.1 macrophages by extract of Polish propolis and its phenolic components: chrysin, galangin, kaempferol, and quercetin [27]. Paulino et al. demonstrated that artepillin C decreases NO concentration in RAW264.7 cells incubated with LPS [46]. Król et al. described downregulation of NO by kaempferide in activated murine peritoneal macrophages [16]. Similar to previous studies, our data confirmed the effect of EEB-P on NO generation in J774A.1 cells. The additional data from *in vitro* study supplied by Tan-No et al. proved that Brazilian propolis extract suppresses corrageenin-induced paw edema in mouse through inhibition of NO production [47].

Macrophages are a major source of many cytokines involved in immune response, hematopoiesis, inflammation, and other homeostatic processes. Upon stimulation by microorganisms, microbial products (e.g., LPS) or endogenous factors (including cytokines), macrophages synthetize *de novo*, and release a large variety of cytokines: IL-1, IL-3, IL-4, IL-5, IL-6, IL-8, IL-9, IL-10, IL-12, IL-13, IL-17, TNF-*α*, IFN-*α*, IFN-*γ*, TGF-*β*, M-CSF, G-CSF, GM-CSF, MCP-1, MCP-3, MCP-5, MIP-1, MIP-2, RANTES, MIF, and KC. In addition, these cytokines can modulate most of the functions of macrophages, cell surface markers expression, and others cytokines secretion. The cytokine network plays a key role in regulation of macrophages activation [48]. To gain further insight into anti-inflammatory activity of EEP-B, nineteen cytokines secreted by macrophages were analyzed. We investigated the influence of Brazilian green propolis extract on production of cytokine IL-1*α*, IL-1*β*, IL-3, IL-4, IL-5, IL-6, IL-9, IL-10, IL-12p40, IL-13, IL-17, TNF-*α*, IFN-*γ*, G-CSF, GM-CSF, MCP-1, MIP-1*α*, MIP-1*β*, and RANTES in LPS + IFN-*γ* stimulated macrophages. Inflammatory cytokines are generated by innate immune cells during infection. For example, LPS induces strong release of IL-1, IL-6, IL-12, and TNF-*α* by the macrophages [37, 48, 49]. The upregulation of these inflammatory cytokines in LPS or LPS + IFN-*γ* activated macrophages was blocked by propolis extracts. We showed that EEP-B significantly downregulated the production of IL-1*α*, IL-1*β*, IL-6, IL-12p40, and TNF-*α* in LPS + IFN-*γ* treated J774A.1 cells. Bachiega et al. described the increase of IL-1*β* and decrease of IL-6 production by Brazilian green propolis extract and its phenolic acids in peritoneal murine macrophages challenged with LPS [50]. Shi et al. and Wang et al. noticed that extracts from Chinese propolis suppress mRNA and protein expression of IL-1*β* and IL-6 induced by LPS in RAW264.7 cells [51, 52]. Blonska et al. reported the inhibition of IL-1*β* mRNA and protein expression in LPS-stimulated J774A.1 macrophages by extract of Polish propolis and its flavones [27]. We observed that the secretion of interleukin IL-4 and IL-13 in LPS + IFN-*γ* activated J774A.1 cells was also reduced by EEP-B. Whereas the concentrations of IL-3, IL-5, IL-9, IL-10, IL-17, and IFN-*γ* were only slightly or not at all affected after incubation with EEP-B. The *in vivo* experiment performed by Franchin et al. showed down-regulation of IL-1*β* and TNF-*α* production by extract of geopropolis from *Melipona scutellaria* [53]. By contrast, Orsatti et al. demonstrated upregulation of IL-1*β* and IL-6 in *in vivo* model [54]. In other *in vivo* test Hu et al. found out that Chinese propolis extracts suppressed carrageenan induced peritonitis and pleurisy or acute lung damage in rats through decrease of the number of neutrophils. Chinese propolis also down-regulated IL-6 in Freund's complete adjuvant (FCA) induced arthritis in rats [55]. Paulino et al. reported a significant inhibition of paw edema in carrageenan induced model of murine peritonitis by artepillin C also *via* decrease of neutrophils' percentage in the peritoneal cavity [46].

The colony-stimulating factors, G-CSF, and GM-CSF release in activated J774A.1 macrophages were decreased by EEP-B. Chemokines (MCP-1, MCP-3, MIP-1, MIP-2, and RANTES) are chemotactic cytokines contributing to the recruitment of circulating monocytes within tissues. Our study extended to macrophage chemokines demonstrated that EEP-B downregulates the expression of MCP-1, MIP-1*α*, MIP-1*β*, and RANTES in LPS + IFN-*γ* stimulated J774A.1 cells. These results suggest a crucial contribution of Brazilian green propolis in the modulation of chemokine-mediated inflammation.

The findings confirm significant anti-inflammatory effect of ethanolic extract of Brazilian green propolis on macrophage *in vitro* model. 

## 5. Conclusion

Propolis has become a subject of special interest as a source of valuable phenolic compounds to developed food components, dietary supplements, or even pharmaceuticals for the prevention or treatment of inflammatory diseases. Artepillin C, drupanin, baccarin, *p*-coumaric acid, and kaempferide are the main ingredients of Brazilian green propolis. Our recent study provide new evidence-based proofs of anti-inflammatory properties of Brazilian green propolis extract.

## Figures and Tables

**Table 1:** The content [mg/g] and characterization of phenolic compounds of the ethanol extract of Brazilian green propolis using their spectral characteristic in negative ions in LC-ESI/MS.

Peak	Retention time *t*_*r*_ (min)	[M−H]^−^	MS/MS fragments	Compound name*	Quantity [mg/g of propolis]**
(1)	1.96	353.0879	191.0553/173.0446/161.0241/135.0440	Caffeoylquinic acid isomer^b,c^	6.49^e^
(2)	2.26	179.0349	161.0241/135.0440	Caffeic acid^a^	1.29
(3)	2.79	163.0406	119.0510	*p*-Coumaric acid^a^	19.90
(4)	3.02	193.0492	193.0492	Ferulic acid^a^	1.48
(5)	3.30	515.1196	353.0879/191.0553/179.0349/161/0241/135.0440	Dicaffeoylquinic acid izomer^b,c^	2.22^e^
(6)	3.46	515.1196	353.0879/191.0553/179.0349/161/0241/135.0441	Dicaffeoylquinic acid izomer^b,c^	2.55^e^
(7)	3.65	231.0652	187.0760/163.0380/145.0653	Coumaric acid prenyl ester^b^	0.14^d^
(8)	3.84	487.1597	263.0911/179.0349/161.0241	Caffeic acid derivative^c^	0.55^e^
(9)	4.27	677.1512	515.1196/353.0879/191.0553/179.0349/161.0241/135.0441	Tricaffeoylquinic acid^b,c^	0.46^e^
(10)	4.43	543.1486	381.1173/179.0349/161.0241	Dimethyl-dicaffeoylquinic acid^c^	3.00^e^
(11)	4.65	543.1486	381.1173/179.0349/161.0241	Dimethyl-dicaffeoylquinic acid^c^	3.61^e^
(12)	4.89	271.0616	151.0017/133.0654/107.0134	Naringenin^a^	0.54
(13)	4.96	285.0394	285.0394	Kempferol^a^	3.35
(14)	5.01	315.0512	301.0709/151.0017	Isorhamnetin^b,c^	3.18^h^
(15)	5.08	301.0709	152.0119/	Hesperitin^a^	8.42
(16)	5.25	301.1455	239.1426/202.0992/132.0568	Ni	—
(17)	5.43	705.1837	543.1486/381.1173/179.0349/161.0241	Caffeic acid derivative^c^	0.83^e^
(18)	5.63	247.0982	203.1072/148.0535	3,4-Dihydroxy 5-prenylcinnamic acid^b^	2.41^d^
(19)	5.71	331.1565	331.1565	Ni	2.75^d^
(20)	5.89	231.1025	187.1124/132.0568	4-Hydroxy 3-prenylcinnamic acid (drupanin)^b^	25.63^d^
(21)	6.05	315.1602	253.1609/201.1286/146.0728	Coumaric acid derivative^c^	3.61^d^
(22)	6.44	285.0775	164.0171/119.0510	Sakuranetin^a^	2.43
(23)	6.46	285.0775	164.0116/151.0042/135.0162/108.0215	Izosakuranetin^a^	3.39
(24)	6.51	255.0663	255.0663/	Pinocembrin^a^	3.12
(25)	6.59	299.0572	284.0313/201.1286/151.0042	Kaempferide^a^	18.81
(26)	6.72	377.1958	299.0572/201.1286	Kaempferide derivative^c^	10.18^g^
(27)	6.78	393.1335	163.0380/145.0284/119.0510	Dicoumaric prenyl ester^c^	3.71^d^
(28)	6.87	315.1602	299.0536/253.1609/198.1039	Ni	4.16^d^
(29)	7.03	313.1433	211.1126/156.0583/149/0618	Ni	0.61^d^
(30)	7.10	529.1495	299.0572/284.0313	Kaempferide derivative^c^	0.07^g^
(31)	7.22	315.1602	201.1286/146.0728	Ni	0.06^d^
(32)	7.42	559.1631	329.0666/163.0380/145.0284/119.0488	Coumaric acid derivative^c^	0.42^d^
(33)	7.49	329.1780	299.1634/255.1742	Artepillin C derivative^c^	—
(34)	7.61	545.2541	331.1900/269.1174	Ni	—
(35)	7.70	477.1917	211.1126/163.0380/145.0284/118.0415	Coumaric acid derivative^c^	0.63^d^
(36)	8.06	299.1634	255.1742/200.1197	Artepillin C^a^	51.44
(37)	8.40	585.1772	301.1455/125.0974	Ni	—
(38)	8.91	447.2171	297.1489/149.0613	Ni	—
(39)	9.13	363.1619	187.1124/149.0613/131.0497	3-Prenyl-4-(dihydrocinnamoyloxy)-cinnamic acid (baccharin)^b^	9.66^d^
(40)	9.32	297.1489	253.1577/198.1039/152.9951	Ni	0.62^d^
(41)	9.51	321.2426	299.1634/255.1742	Artepillin derivative^c^	0.89^e^
(42)	9.77	321.2426	299.1634/255.1742	Artepillin derivative^c^	2.99^e^
(43)	10.99	613.3177	299.1634/281.1550/154.1682	Artepillin derivative^c^	1.41^e^

Ni: not identified.

^∗,a^Confirmed by standard.

^b^Confirmed by reference [19–21, 23, 24, 26].

^c^Confirmed by MS fragmentation.

^∗∗,d^Expressed as *p*-coumaric acid.

^e^Expressed as caffeic acid.

^f^Expressed as artepillin C.

^g^Expressed as kaempferide.

^h^Expressed as rhamnetin.
